# Extracts and Terpenoids from *Stevia* Species as Potential Anthelmintics for Neglected Tropical Diseases Caused by Cestode Parasites

**DOI:** 10.3390/molecules29184430

**Published:** 2024-09-18

**Authors:** María del Pilar Cevasco Contreras, Jimena Borgo, Ana María Celentano, Orlando Germán Elso, Hernán Bach, Cesar Atilio Nazareno Catalán, Augusto Ernesto Bivona, Hugo Rolando Vaca, Mara Cecilia Rosenzvit, Valeria Patricia Sülsen

**Affiliations:** 1 Departamento de Microbiología, Facultad de Medicina, Universidad de Buenos Aires, Buenos Aires C1113AAD, Argentina; pilarcevasco@gmail.com (M.d.P.C.C.); amcelentano@yahoo.com.ar (A.M.C.); hugorvaca@gmail.com (H.R.V.); 2 Instituto de Investigaciones en Microbiología y Parasitología Médica (IMPaM) (UBA-CONICET), Universidad de Buenos Aires, Paraguay 2155, Piso 13, Buenos Aires C1113AAD, Argentina; 3 Instituto de Química y Metabolismo del Fármaco (IQUIMEFA) (UBA-CONICET), Universidad de Buenos Aires, Junín 956, Piso 2, Buenos Aires C1113AAD, Argentina; jborgo@docente.ffyb.uba.ar; 4 Cátedra de Farmacognosia, Facultad de Farmacia y Bioquímica, Universidad de Buenos Aires, Junín 956, Piso 2, Buenos Aires C1113AAD, Argentina; orlando.elso@gmail.com; 5 Unidad de Microanálisis y Métodos Físicos Aplicados a Química Orgánica (UMYMFOR) (UBA-CONICET), Ciudad Universitaria, Pabellón 2, Piso 3, Buenos Aires C1428EGA, Argentina; 6 Instituto Nacional de Tecnología Agropecuaria, Nicolas Repetto y De los Reseros s/n, Hurlingham, Buenos Aires B1686IQN, Argentina; bach.hernan@inta.gob.ar; 7 Instituto de Química Orgánica, Facultad de Bioquímica, Química y Farmacia, Universidad Nacional de Tucumán, Ayacucho 471, San Miguel de Tucumán T4000INI, Argentina; cancatalan@gmail.com; 8 Instituto de Estudios de la Inmunidad Humoral (IDEHU) (UBA-CONICET), Junín 956, Piso 4, Buenos Aires C1113AAD, Argentina; augustobivona@gmail.com; 9 Institut de Génétique et de Biologie Moléculaire et Cellulaire, Université de Strasbourg, CNRS, INSERM, UMR 7104, U 1258, 67404 Illkirch, France

**Keywords:** natural bioactive compounds, medicinal plants, *Stevia*, neglected tropical parasitic diseases, *Echinococcus*, anti-parasitic treatment

## Abstract

Cestodes are etiological agents of neglected diseases such as echinococcosis and cysticercosis, which are major public health problems. Antiparasitic treatment relies on a small number of approved drugs, which are often only partially effective, poorly tolerated and require prolonged administration. Thus, the discovery of novel potential treatments is critical. The *Stevia* genus (Asteraceae) includes species that are recognized as a source of bioactive compounds, with many species associated with medicinal uses. In this study, the cestocidal activity of four South American *Stevia* species that previously showed antiprotozoal activity was analyzed using a motility assay on the laboratory cestode model, *Mesocestoides vogae*. The four *Stevia* extracts showed cestocidal activity, with *S. alpina* var. *alpina* as the most active. The sesquiterpene lactones estafietin and eupatoriopicrin were purified from *S. alpina* var. *alpina* and *S. maimarensis*, respectively, and tested on *M. vogae*. Estafietin showed cestocidal activity, inhibiting parasite viability in a dose-dependent manner, even from the first day of incubation. Consistent with the motility effects, the extract of *S. alpina* var. *alpina* and estafietin induced marked alterations in the morphology of the parasite. The results of this report show that *Stevia* species represent a source of new molecules with potential for the treatment of neglected tropical diseases caused by cestodes.

## 1. Introduction

Cestodes, or tapeworms, are an ample and diversified group of obligate endoparasites able to infect many animal species worldwide [1]. Several members of the class Cestoda are of medical importance since they use humans and other animals as definitive and/or intermediate hosts, causing severe diseases. Among them, *Echinococcus granulosus sensu lato* and *Echinococcus multilocularis* are the etiological agents of cystic and alveolar echinococcosis, respectively, and *Taenia solium* causes cysticercosis [2]. These diseases are among the 20 neglected tropical diseases prioritized by the World Health Organization, which mainly affect people living in poverty principally in tropical and subtropical regions [3]. 

Currently, the chemotherapeutic options to treat echinococcosis and cysticercosis are very limited, relying mainly on the benzimidazoles albendazole and mebendazole, as well as praziquantel. Benzimidazoles are heterocyclic aromatic compounds formed by the fusion of benzene and imidazole rings. Their primary mode of action is selective binding to helminth β-tubulin, inhibiting its polymerization and the formation of microtubules, hampering glucose uptake and other cellular functions [4]. Praziquantel is a derivative of pyrazinoquinoline. The mechanism of action of praziquantel is not completely defined. It is known that praziquantel induces high rates of calcium ion influx, resulting in uncontrolled muscle contraction and paralysis of the worms. The postulated drug targets that trigger this calcium ingress are voltage-gated calcium channels [5], or more recently, a transient receptor potential melastatin ion channel (TRPM) [6]. These drugs present some disadvantages in terms of safety, tolerance, efficacy, length of treatment and access for the affected population. Approximately 20–40% of inefficacy was reported for benzimidazoles in treating cystic echinococcosis [7,8], and 60% of treatment failures were estimated for alveolar echinococcosis [9]. Albendazole is parasitostatic only, especially for *E. multilocularis*, implying that long-term treatment, often life-long, with concomitant adverse effects is needed to treat alveolar echinococcosis. Currently, the availability of these drugs is low, and the cost is high in many endemic countries [10]. This scenario highlights the need for new, effective, safe and affordable drugs to control these cestodiases.

One of the main limitations of research on cestodes is the low availability of parasitic material. Some zoonotic cestodes, such as *E. granulosus sensu lato*, cannot be maintained in experimental models, being obtained only from natural infections, which makes the implementation of systematic drug evaluation studies difficult. *Mesocestoides vogae* is a cestode whose tetrathyridium (TTy) larval stage can multiply continuously in laboratory animals, providing a reproducible source of material for biological assays. Furthermore, it is easily cultivated in vitro and is considered non-zoonotic, so it can be handled safely [11,12]. This parasite has been validated as a laboratory model [13], being used for development studies [14,15,16], and for the identification of new cestocidal compounds in pharmacological studies [17,18].

Natural products have made a significant contribution to the development of medicines against a variety of pathologies. It is estimated that more than 50% of the drugs currently on the market are derived from natural sources or were inspired by nature [19]. In this sense, natural products represent an extensive source of molecules. Out of 300,000 plant species inhabiting the Earth, only 6% have been investigated for their pharmacological properties. Furthermore, the molecular complexity and diversity of these compounds, inherent to the biosynthetic machinery of a living organism, are incomparable to the structural possibilities of synthetic molecules. Molecular complexity is often accompanied by highly selective and specific biological activities, as seen in cases such as taxol and artemisinin [20]. These properties make natural products highly attractive for the discovery of lead molecules in drug design.

The *Stevia* genus (Asteraceae) consists of more than 230 plant species, mainly distributed from the Southern United States to the South American Andean region. *Stevia rebaudiana* Bertoni is the most popular member of this genus since it is known to produce the diterpene glycoside sweetener stevioside. Beyond *S. rebaudiana*, numerous other species belonging to the *Stevia* genus are recognized for their medicinal properties [21]. Ethnobotanical records of *Stevia* are described in the book *Natural History of Plants of the New Spain*, written between 1570 and 1576 by Francisco Hernandez [22]. *Stevia* species have been popularly employed to treat various ailments since the 18th century [21]. Popularly, drinks such as decoctions and infusions have been used as febrifuges, to treat inflammation, to cure wounds and for intestinal upsets due to parasites, among other uses [22].

The *Stevia* genus is characterized by the presence of sesquiterpene lactones, diterpenes, longipinanes and flavonoids. Extracts and compounds isolated from these plant species have demonstrated diverse pharmacological activities, including antioxidant, antiviral, anti-inflammatory, and antiproliferative properties [23]. Furthermore, antiparasitic activity of several *Stevia* extracts and different compounds obtained from plants of this genus has been reported, including the phytochemicals eupatorin, estafietin and eupatoriopicrin, isolated from *S. satureiifolia* var. *satureiifolia*, *S. alpina* var. *alpina* and *S. maimarensis*, respectively, which exerted trypanocidal activity [24,25,26]. On the other hand, the extracts of *S. satureiifolia* var. *satureiifolia*, *S. entreriensis*, *S. multiaristata* and *S. aristata* have demonstrated trypanocidal, leishmanicidal and/or anti-*Echinococcus granulosus* activities [27,28,29].

Considering the potential of natural products in the drug discovery process and the antiparasitic activity demonstrated by some species of the *Stevia* genus, this work aimed to analyze the effect of *Stevia* extracts and isolated compounds on *M. vogae* in the search for selective and effective drugs to treat neglected tropical diseases caused by cestodes.

## 2. Results

### 2.1. Stevia Extracts and Pure Phytochemicals

**Figure 1:**
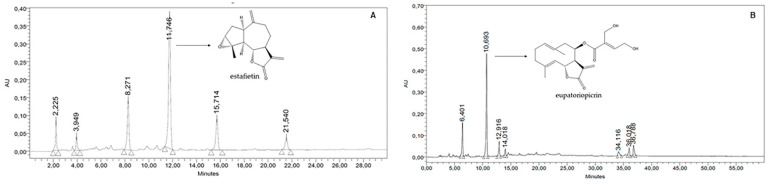
HPLC-DAD chromatograms of the organic crude extract of *Stevia alpina* var. *alpina* (**A**) and *Stevia maimarensis* (**B**).

The dichloromethane extracts from the Argentinian species *S. alpina* var. *alpina*, *S. multiaristata*, *S. maimarensis* and *S. aristata* were prepared by maceration at room temperature. The extraction process resulted in yields of 5.0, 16.6, 5.3 and 2.5%, respectively, referring to the dried plant. The chromatographic analysis of the crude extracts of *S. alpina* var. *alpina* and *S. maimarensis* was performed by HPLC coupled with a UV-diode array detector (HPLC-DAD). The chromatograms obtained are shown in Figure 1.

The dichloromethane crude extract of *S. alpina* var. *alpina* showed nine peaks. A major peak of 52.43% of the total peak area was observed at a retention time (Rt) of 11.746 min (Figure 1A). This compound showed a UV spectrum with a maximum absorption (λmax) at 210 nm, corresponding to the sesquiterpene lactone estafietin (compound **1**). The dichloromethane crude extract of *S. maimarensis* showed seven peaks, with one major peak that represented 53.35% of the total peak area at a Rt of 10.693 min (Figure 1B). The UV spectrum of this peak presented a λmax at 212 nm. This peak corresponds to the sesquiterpene lactone eupatoriopicrin (compound **2**).

**Figure 2:**
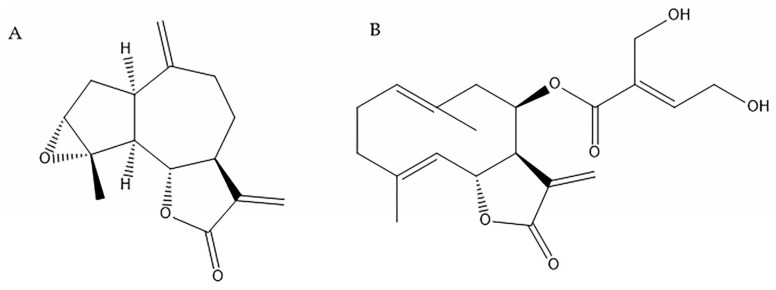
Chemical structures of the sesquiterpene lactones estafietin (**A**) and eupatoriopicrin (**B**) isolated from *Stevia alpina* var. *alpina* and *Stevia maimarensis*, respectively.

The organic extracts of *S. alpina* var. *alpina* and *S. maimarensis* were processed as detailed in Section 4.4 in order to isolate the sesquiterpene lactones estafietin and eupatoriopicrin, respectively. These compounds were identified by spectroscopic methods comparing the experimental spectra with those found in the literature [30,31]. The chemical structures of estafietin and eupatoriopicrin can be seen in Figure 2. The purity levels of these phytochemicals determined by HPLC-DAD were 93.3 and 94.6%, respectively.

### 2.2. Effect of Stevia Extracts and Compounds on Cestode Viability

**Figure 3:**
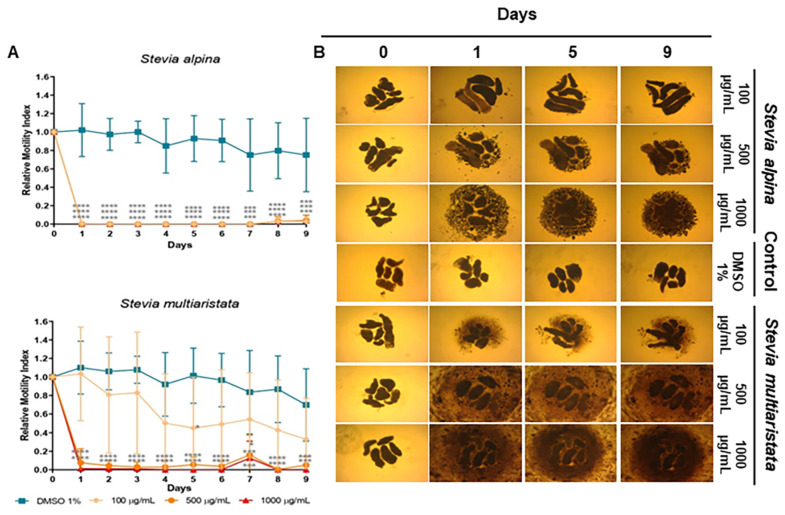
Effect of *Stevia* extracts in *Mesocestoides vogae* tetrathyridia. (**A**) In vitro cestocidal activity determined by worm motility for 9 days using a worm tracker device for the extracts: *Stevia alpina* var. *alpina* and *Stevia multiaristata*. Relative motility indices were measured from three independent biological replicates, each one in quadruplicate. Error bars represent the standard deviation, and the asterisks indicate those values that showed differences with statistical significance compared with the negative control, according to the two-way ANOVA test and Dunnett’s post-tests (* *p* < 0.05, *** *p* < 0.001; **** *p* < 0.0001). (**B**) Inverted optical microscope images (40×) of *S. alpina* var. *alpina* and *S. multiaristata* extracts in *M. vogae* tetrathyridia on different days of treatment. The extracts were evaluated at concentrations of 100, 500 and 1000 µg/mL. Parasites incubated with the drug vehicle (DMSO 1%) were used as a negative control. Morphological alterations and extensive damage to the tegument are observed.

**Figure 4:**
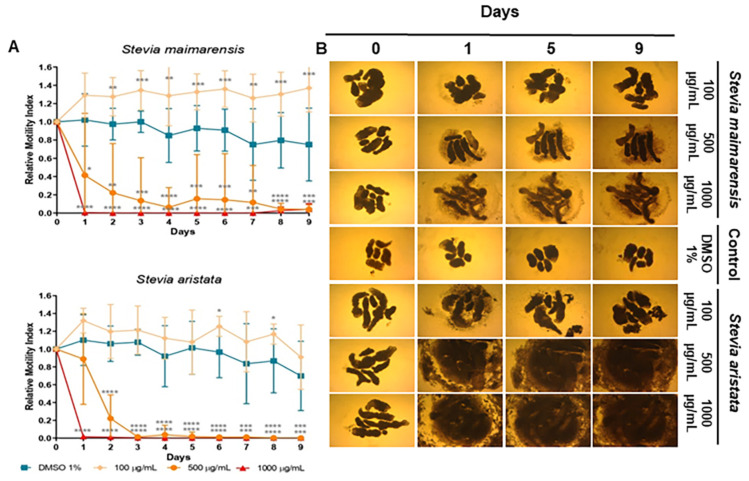
Effect of *Stevia* extracts in *Mesocestoides vogae* tetrathyridia. (**A**) In vitro cestocidal activity determined by worm motility for 9 days using a worm tracker device for the extracts: *Stevia maimarensis* and *Stevia aristata*. Relative motility indices were measured from three independent biological replicates, each one in quadruplicate. Error bars represent the standard deviation, and the asterisks indicate those values that showed differences with statistical significance compared with the negative control, according to the two-way ANOVA test and Dunnett’s post-tests (* *p* < 0.05; ** *p* < 0.01; *** *p* < 0.001; **** *p* < 0.0001). (**B**) Inverted optical microscope images (40×) of *S. maimarensis* and *S. aristata* extracts in *M. vogae* tetrathyridia on different days of treatment. The extracts were evaluated at concentrations of 100, 500 and 1000 µg/mL. Parasites incubated with the drug vehicle (DMSO 1%) were used as a negative control. Morphological alterations and extensive damage to the tegument are observed.

As detailed in Section 4.9, the cestocidal activity of *Stevia* species extracts and compounds was evaluated with a worm tracker device that quantifies *M. vogae* TTy motility. Additionally, the alterations on TTy structures were recorded daily with a camera attached to an inverted optical microscope. *Stevia alpina* var. *alpina* extract showed the highest cestocidal activity of the four species analyzed here. This extract showed an almost complete reduction of viability from day 1 at all concentrations tested (96–100%, *p* < 0.0001 days 1–9 for all concentrations) (Figure 3A). The other three extracts showed a dose-dependent response. At a high concentration (1000 μg/mL), they killed TTy from day 1 (96–100%, *p* < 0.001–*p* < 0.0001, days 1–9) (Figure 3A and Figure 4A). *S. multiaristata* showed a high cestocidal activity also at 500 μg/mL (84–97%, *p* < 0.001–*p* < 0.0001, days 1–9) (Figure 3B). *S. maimarensis* and *S. aristata*, showed similar cestocidal activity. At 500 μg/mL, an initial reduction of viability was observed (*S. maimarensis*: 59%, *p* < 0.05, day 1; 78%, *p* < 0.01, day 2; *S. aristata*: 78%, *p* < 0.0001, day 2) and a delayed high cestocidal action (days 3–9: *S. maimarensis*: 84–96%, *p* < 0.01–*p* < 0.001; *S. aristata*: 96–100%, *p* < 0.001–*p* < 0.0001). An unexpected stimulatory effect was displayed at 100 μg/mL (*S. maimarensis*: 26–37% of increase, *p* < 0.01–*p* < 0.001, days 2–9) (Figure 4A) (*S. aristata*: 8% of increase on days 6 and 8, *p* < 0.05) (Figure 4A).

**Figure 5:**
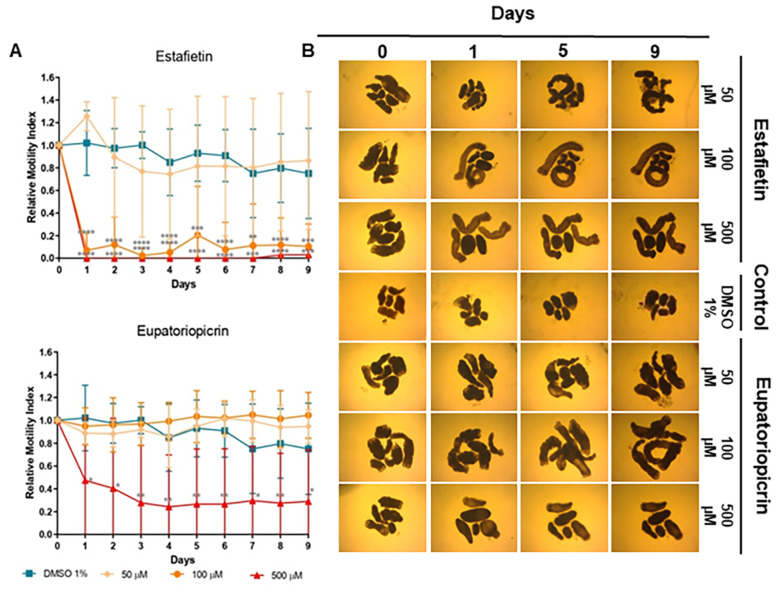
Effect of estafietin and eupatoriopicrin in *Mesocestoides vogae* tetrathyridia. (**A**) In vitro cestocidal activity determined by worm motility for 9 days using a worm tracker device for the compounds: Estafietin and Eupatoriopicrin. Relative motility indices were measured from three independent biological replicates, each one in quadruplicate. Error bars represent the standard deviation, and the asterisks indicate those values that showed differences with statistical significance compared with the negative control, according to the two-way ANOVA test and Dunnett’s post-tests (* *p* < 0.05; ** *p* < 0.01; *** *p* < 0.001; **** *p* < 0.0001). (**B**) Inverted optical microscope images (40×) of estafietin and eupatoriopicrin in *M. vogae* tetrathyridia on different days of treatment. The compounds were evaluated at concentrations of 50, 100 and 500 µM. Parasites incubated with the drug vehicle (DMSO 1%) were used as a negative control. Morphological alterations and extensive damage to the tegument are observed.

Estafietin, the main compound of *S. alpina* var. *alpina*, induced a strong dose-dependent reduction of viability at 500 (97–100%, *p* < 0.001–*p* < 0.0001) and 100 μM (79–97%, *p* < 0.001–*p* < 0.0001), with no effect at 50 μM (Figure 5A). Eupatoriopicrin was less effective than estafietin. This compound, obtained from *S. maimarensis*, showed a delayed reduction of viability at 500 μM (53%, day 1; 60%, day 2; 71–76%, days 3–9; *p* < 0.05–*p* < 0.01). Unlike the parent extract, which produced an 8% increase in viability, TTy treated with lower concentrations of this terpene displayed similar values as negative control parasites (Figure 5A).

The results of microscopical observations confirmed those obtained with the WMicrotracker. An evident alteration of general morphology (Figure 3B, Figure 4B and Figure 5B), together with a lack of motility, was observed in treated TTY on the inverted microscope at the same concentrations of extracts and compounds with no registers of motor activity with the WMicrotracker.

**Figure 6:**
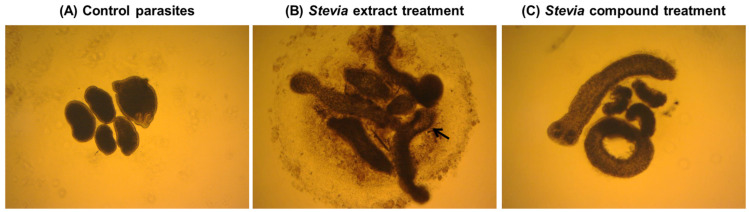
Inverted optical microscope images (40×) showing the main morphology alterations observed after *Stevia* extracts and purified compounds treatment. (**A**) Parasites incubated in MvRPMI medium with the drug vehicle (DMSO 1%) used as a negative control, day 5 of treatment. (**B**) Parasites incubated in MvRPMI medium with *Stevia maimarensis* extract at a concentration of 1000 μg/mL, day 5 of treatment. (**C**) Parasites incubated in MvRPMI medium with estafietin at a concentration of 100 μM, day 5 of treatment. Note the loss of definition of the tegument surrounding the parasites in (**B**,**C**) compared to the negative control (**A**). Also note an elongation of the body of the parasites in (**B**,**C**) in comparison to (**A**). The black arrow indicates an influx of the culture medium in the parasite parenchyma.

Debris of the plant material made the observations more difficult and prevented evaluations of tegumental debris, with alterations observed better at low concentrations of extracts and TTY treated with purified compounds. Details of the main morphology alterations are indicated in Figure 6.

The main TTY structure alterations induced by *S. alpina* var. *alpina*, *S. maimarensis* and *S. aristata* were an elongation of the body and a loss of tegumental definition with some parasites with a pale aspect (Figure 3B, Figure 4B and Figure 6B). In some cases, an influx of the culture medium, related to the tegumental damage, was observed as a void space in the parasite parenchyma (Figure 6B). Similar morphological alterations were observed with estafietin and eupatoriopicrin (Figure 5B and Figure 6C). TTys treated with *S. multiaristata* looked denser and more compact than those treated with the other extracts and also exhibited extensive damage in the tegument (Figure 3B).

## 3. Discussion

The scarcity of safe, effective and affordable drugs to treat diseases caused by cestodes such as echinococcosis and cysticercosis, which mainly affect vulnerable populations, highlights the importance of finding new treatment alternatives. Previous works assessed the effect of plant extracts from the Asteraceae family on *E. granulosus sensu stricto* (s.s.) protoscoleces and cysts obtained from the murine experimental model and observed that the extracts from *S. aristata* [28] and *S. multiaristata* [29] showed in vitro protoscolicidal potential. Both *Stevia* extracts also caused damage to the germinal layer of murine cysts and produced a significant reduction in the parasitic mass obtained from mice infected with *E. granulosus* s.s. protoscoleces. Based on these findings, the cestocidal potential of the mentioned *Stevia* extracts and two additional ones from *S. alpina* var. *alpina* and *S. maimarensis* were evaluated in our *M. vogae* model. *Stevia alpina* var. *alpina* showed the highest anthelmintic potential followed by *S. multiaristata* and *S. maimarensis*. In view of these results, the major compounds of these extracts were isolated and their effect on *M. vogae* TTY was evaluated. The *S. multiaristata* extract could not be processed due to the low amount of plant material available.

The cestocidal effect of the compounds was measured by quantifying worm motility using the worm microtracker device [32] adapted to cestodes [33,34,35,36]. This method allows the simultaneous evaluation of a high number of compounds, objectively and quantitatively. It also enables continuous, real-time and non-invasive measurements, facilitating the evaluation of parasitic viability on the same plate throughout the testing period. *M. vogae* is a cestode laboratory model that allows the implementation of systematic drug evaluation studies. The results obtained showed that all the tested extracts produced a high and significant reduction of *M. vogae* TTy viability at the highest concentration tested (1000 µg/mL). At lower concentrations, *S. alpina* var. *alpina* was the most potent, reducing parasite viability by more than 95% from the first day of incubation.

Estafietin, the major compound present in *S. alpina* var. *alpina* extract, showed a high cestocidal potential, being able to kill 100% of parasites at the highest concentration tested −500 µM, and more than 76% of parasites at 100 µM, even from the first day of incubation. On Vero cells, this compound presented a 50% cytotoxicity concentration (CC_50_) value of 800.8 µM [36], thus indicating it does not show overt toxicity against this human cell line. The early effect of this compound suggests that it could help shorten cestocidal treatments. This is of great importance considering the long-term treatment of echinococcosis using albendazole. A delayed effect for albendazole at 20 µM was also shown by our previous studies [36]. Eupatoriopicrin, isolated from *S. maimarensis* extract, showed cestocidal capacity although it was less potent and selective than estafietin (CC_50_ = 257.7 µM) [37]. This compound reduced parasite motility or did not modify it, depending on the concentration used. It did not increase parasite motility, suggesting that the low increase in motility observed with *S. maimarensis* could be due to other compounds present in the extract.

The morphology of *M. vogae* TTy larvae, when obtained from the mouse peritoneal cavity, consists of an anterior scolex with four suckers as attachment organs; an internal region with compact, acoelomate and non-segmented parenchyma; and a well-defined external syncytial (multinucleate) tegument that covers the body [38]. The main morphological alterations caused by *Stevia* extracts and compounds were an alteration of tegument definition and elongation of the body.

Both estafietin and eupatoriopicrin are sesquiterpene lactones (STLs), which are a class of terpenoid compounds mainly found in species of the Asteraceae family. STLs have been thoroughly studied due to their wide range of biological activities. These activities include antimicrobial, antitumor, anti-inflammatory, molluscicidal, antihelminthic, antiprotozoal, among others [31]. Moreover, STLs play a crucial role in plant–insect interactions, serving as attractants, deterrents and antifeedants. Estafietin and eupatoriopicrin have an exomethylene ɣ-lactone ring and would react with sulfhydryl groups of enzymes and proteins by Michael-type addition. The guaianolide estafietin also has an epoxy group that could confer more reactivity to the molecule. This was evidenced by the cestocidal activity shown by estafietin in comparison to eupatoriopicrin. Several studies investigated the anthelmintic potential against *E. granulosus* of plant extracts or essential oils. However, only a few of them have focused on the effect of isolated compounds from plants [39]. Concerning STLs, some studies of their effect on *E. multilocularis* were reported. Since dihydroartemisinin and artesunate were effective against *E. multilocularis* metacestodes in vitro but not in vivo (mouse model), the in vitro effects of synthetic ozonides (1,2,4-trioxolanes) were investigated. These compounds were shown to induce structural alterations in the parasites [40]. No further reports of the effects of STLs on cestodes were found in the literature.

Herein, estafietin and its parent extract of *S. alpina* var. *alpina* produced an early and potent in vitro cestocidal effect. In the future, experiments will be carried out to evaluate their in vivo effect in animal models. The results of this work suggest that sesquiterpene lactones, terpenoid compounds present in Asteraceae, such as *Stevia* species, could be considered potential candidates for the development of new medicines to treat neglected diseases caused by cestode parasites alone or in combination with currently used drugs.

## 4. Materials and Methods

### 4.1. Plant Materials

The aerial parts of the *Stevia* species used in this work were collected from different locations within Argentine territory. In order to preserve the genetic resources and the natural ecosystem, these plant materials were harvested conservatively, cutting 10–15% of the aerial parts with scissors [26]. *Stevia maimarensis* (Hieron.) Cabrera was collected in Jujuy Province, Tilcara Department: Perchel, in March 2017. *Stevia alpina* Griseb. var. *alpina* was collected in Tucumán Province, Provincial Route 307, Km 49, in April 2015. *Stevia aristata* D. Don ex Hook. & Arn. was collected in Entre Ríos Province, La Paz Department, Provincial Route 1, Esquivel stream, in December 2012. *Stevia multiaristata* Spreng. was collected in Entre Ríos Province, Paraná Department, Pueblo Brugo, Paraná River in December 2012. The renowned taxonomists Dr. Gustavo Giberti and Hernan Bach conducted the identification of the species. Voucher specimens are available at the Museo de Farmacobotánica “Juan A. Domínguez”, Facultad de Farmacia y Bioquímica, Universidad de Buenos Aires, Argentina, under the identification numbers BAF12264, BAF12266, BAF797 and BAF798, respectively.

The plant materials collected from each species were dried at room temperature protected from sunlight and humidity and afterward manually grounded to be stored until used.

### 4.2. Extract Preparation

Once dried and grounded, fifty grams of *S. alpina* var *alpina* and *S. maimarensis*, ten grams of *S. aristata* and five grams of *S. multiaristata* were extracted to obtain the crude organic extract from each species. Consequently, the material was extracted twice by maceration with dichloromethane (Sintorgan, Buenos Aires, Argentina) (CH_2_Cl_2_) (10% *w*/*v*) for 5 min. The resulting extracts were filtered (grade 0859, medium, smooth, 90 mm, Schleicher and Schuell-Whatman, Buckinghamshire, UK) and taken to dryness at 40°C under vacuum in a rotary evaporator (R-134, Buchi, Flawil, Switzerland). The yield of the extraction process was calculated as yield (%) = (weight of the obtained extract (g) × 100)/weight of initial plant material (g).

### 4.3. Chromatographic Analysis of the Stevia Crude Extracts

The chromatographic analysis of the crude extracts *S. alpina* var. *alpina* and *S. maimarensis* was performed by high-performance liquid chromatography (HPLC) with a Waters equipment (Milford, CT, USA) coupled to a photodiode array detector (Waters 2996), a Rheodyne injection valve (20 μL), pump (Waters Delta 600, Milford, MA, USA), Waters 600 controller and in-line degasser. A reversed-phase column (Agilent Eclipse Plus C-18, 4.6 × 250 mm, 5 μm particle size, Agilent, Santa Clara, CA, USA) was used, and the photodiode array detector was set at 210 nm. The extracts were dissolved in water: acetonitrile (1:1) at 10 mg/mL concentration. The solutions were filtered with a nylon filter (0.45 µM, Agilent, Santa Clara, CA, USA) and eluted with a gradient of water (A) and acetonitrile (B) in 40 min. The gradient used was 35–95% B for both extracts. The flow rate was 1.0 mL/min, and the elution was performed at room temperature. Chromatograms were recorded and processed using the Empower Pro 3 software (https://www.waters.com/waters/en_US/Empower-3-Chromatography-Data-Software/nav.htm?cid=513188&locale=en_US, accessed on 10 September 2024). The water employed to prepare the mobile phase was of ultrapure quality (Milliq). Acetonitrile (HPLC) J. T. Baker and methanol (HPLC) J. T. Baker (Phillipsburg, KS, USA) were used.

### 4.4. Isolation and Purification of Phytochemicals

The crude extract of *S. alpina* var. *alpina* (1.5 g) was fractionated by Silicagel 60 column chromatography (40 × 3 cm, 80 g, 230–400 mesh, Macherey-Nagel, Dueren, Germany) eluted with CH_2_Cl_2_: ethyl acetate (EtOAc, Sintorgan, Argentina) (9.5:0.5). Fractions of 75 mL each were collected and tested by TLC (SP: Silicagel F_254,_ Merck, Darmstadt, Germany; MP: CH_2_Cl_2_:EtOAc (9.5:0.5); SR: sulfuric anisaldehyde). From fractions 3–6, a major compound precipitated in the form of white sharp needles. This precipitate was dissolved in the minimum volume of a heated mixture of heptane: EtOAc (2:1) to be cooled to 4 °C for 24 h in order to facilitate the crystallization process. Afterward, the crystals were separated from the solution and dried under a vacuum to afford compound **1**.

The crude extract of *Stevia maimarensis* (8.1 g) was submitted to a dewaxing process to eliminate sterols and lipids. The extract obtained as described in Section 2.2 was suspended in 200 mL of ethanol:water (70:30) to be partitioned three times with 60 mL of hexane (Sintorgan, Villa Martelli, Argentina). The remaining hydroalcoholic suspension was extracted thrice with 60 mL of CH_2_Cl_2_. The resulting dichloromethane sub-extracts were gathered, dried with anhydrous sodium sulfate (Biopack, Ciudad Autónoma de Buenos Aires, Argentina), filtered and taken to dryness under reduced pressure. The dewaxed extract of *S. maimarensis* (10 g) was fractionated by column chromatography (50 × 5 cm) using Silicagel 60 (180 g, 230–400 mesh, Macherey-Nagel, Dueren, Germany) as the stationary phase (SP) and CH_2_Cl_2_:EtOAc (1:2) as the mobile phase (MP). A total of 40 fractions of 50 mL each were collected. The fractions were tested by Thin Layer Chromatography (TLC) [SP: Silicagel F_254_ (Merck, Germany); MP: Hx:EtOAc (5:5); SR (spraying reagent): sulfuric anisaldehyde, Sigma-Aldrich, St. Louis, CA, USA]. Between fractions 17 and 29, one pure compound was detected; these fractions were gathered and taken to dryness under reduced pressure to afford yellow crystals corresponding to compound **2**.

### 4.5. Purity Assessment and Identification of the Isolated Compounds

The purity analysis of compounds **1** and **2** was carried out using HPLC. A Waters (USA) chromatograph equipped with a UV-visible diode array detector (Waters 2996) and a pump (Waters Delta 600) was utilized. An Agilent Eclipse Plus C-18 analytical column of 4.6 × 250 mm and 5 μm particle size (Agilent, USA) was employed. Gradients of water:acetonitrile (A:B) were used as MP (45–95% B and 35–95% B, for compounds **1** and **2**, respectively.) The compounds were dissolved in a mixture of A:B (1:1) using an ultrasonic bath. Subsequently, the solutions were filtered using 0.45 μm nylon filters. In all cases, a 20 μL loop and a flow rate of 1 mL/min were used. The runtime was set to 30 min. A run for the solvent was performed by injecting the solvent mixture used to dissolve the compounds. The purity was calculated as purity (%) = (area of the major peak × 100)/∑ area of every peak.

The identities of the isolated compounds were determined by spectroscopic methods: proton nuclear magnetic resonance (^1^H-NMR) and carbon nuclear magnetic resonance (^13^C-NMR), heteronuclear single quantum correlation (HSQC), heteronuclear multiple bond correlation (HMBC), correlated spectroscopy (COSY) (Bruker Avance 600, Billerica, MA, USA) (600 MHz in CDCl3), electron impact mass spectrometry (EI-MS) and spectrophotometry (UV), comparing experimental spectra with literature data [30,31].

### 4.6. Drugs Preparation for Biological Assays

Stock solutions of the isolated *Stevia* compounds and the crude extracts were prepared using dimethyl sulfoxide (DMSO, Merck, Darmstadt, Germany) as the vehicle at concentrations of 30 mg/mL and 100 mg/mL, respectively. The solutions were then fractionated and stored at −18 °C and thawed in an ultrasonic bath at 40 °C to later be diluted as necessary for each biological assay.

### 4.7. Ethics Statement Assays

Experiments involving the use of experimental animals were conducted strictly in accordance with the protocols approved by the Comité Institucional para el Cuidado y Uso de Animales de Laboratorio (CICUAL), Facultad de Medicina, Universidad de Buenos Aires (UBA), Argentina (protocols: “In vivo passages of cestode parasites from *Mesocestoides vogae*” CD Nº 1127/2015 and “Histone modifying enzymes in flatworms: study of their potential as new drug targets in diseases of importance in veterinary medicine and human health” CD Nº 187/2020).

### 4.8. Parasite Material

The *M. vogae* tetrathyridia (TTy) used in this work were maintained in the laboratory by alternate intraperitoneal infection in Wistar rats and BALB/c mice, as described previously [41]. The experimental animals were bred and housed in a temperature-controlled light cycle room with food and water *ad libitum* at the animal facilities of Instituto de Investigaciones en Microbiología y Parasitología Médica (IMPaM), Universidad de Buenos Aires (UBA) and Consejo Nacional de Investigaciones Científicas y Técnicas (CONICET), Ciudad Autónoma de Buenos Aires, Argentina. After 3 months of infection, mice were euthanized by CO_2_ inhalation. TTy were collected from the peritoneal cavity, using standard aseptic techniques, and washed three times with sterile PBS solution, pH 7.2. Finally, before being employed in experiments, TTy were size-selected using monofilament polyester meshes to a final size of 150–250 µm and incubated for 24 h in 5 mL of MvRPMI medium, a modified RPMI 1640 medium without phenol red (Sigma-Aldrich, St. Louis, USA) complemented with 10% *v/v* inactivated fetal bovine serum (INTERNEGOCIOS SA, Mercedes, Argentina), 2.4 g/L of HEPES Free acid (JT Baker, USA), 2.5 g/L of glucose (4.5 g/L final concentration, Britania, Ciudad Autónoma de Buenos Aires, Argentina), 2 g/L of Sodium Bicarbonate (Anedra, Ciudad Autónoma de Buenos Aires, Argentina) and 1% *v/v* Pen/Strep (Penicillin-Streptomycin 10,000 U/mL, Gibco, Cambridge, MA, USA) at 37 °C under 5% CO_2_ atmosphere.

### 4.9. In Vitro Anthelmintic Assays

The in vitro effect of each extract and compound on parasite viability was studied as described before by our group in Facultad de Medicina [33,34,35,36] with minimum modifications. Viability was evaluated with a motility assay employing a worm tracker device (WMicrotracker MINI, Designplus SRL, Sunchales, Argentina) [32] with an algorithm adapted to measure the movement of *M. vogae* TTy [33,34,35,36]. Briefly, the 24 h incubated TTy, as described in 4.8, were distributed in U-shaped 96-well microplates (Greiner Bio-One, Frickenhausen, Germany) (five TTy per well) with 150 µL of MvRPMI medium at 37 °C under 5% CO_2_ atmosphere and incubated for an additional 24 h. Incubations with extracts or compounds were performed the following day. Extracts were tested at concentrations of 100, 500 and 1000 µg/mL and pure compounds at concentrations of 50, 100 and 500 µM. As positive controls, pre-treated parasites with ethanol 70% (Anedra, Argentina) for 30 min and the antiparasitic drug praziquantel (Sigma-Aldrich, USA) at 20 µM were used. All motility assays were performed using an equal amount of the drug vehicle (1% DMSO final concentration, Thermo Scientific, Waltham, MA, USA) and the corresponding negative control (1% DMSO). To determine the effects of the treatments, TTy were incubated for nine days without changing the medium in the same conditions as described above. Measurements of motility with the WMicrotracker and microscopical observations were performed before adding the compounds (day 0) and daily afterward. The data was collected from three independent biological replicates, each corresponding to TTy obtained from a different mouse, in quadruplicate for each tested condition. Relative motility indices (RMI) of each well respective to its motility before adding the compounds were calculated as described previously [33,34,35,36].

Statistical analyses on motility assays were carried out as in previous studies [33,34,35,36]. GraphPad Prism 8.0.2 software (GraphPad Software Inc., Dotmatics, Boston, MA, USA) was employed for the analysis. Repeated two-way ANOVA tests were used to analyze the effects of the compounds on TTy motility. Significant differences (*p* < 0.05) were determined by Dunnett’s comparisons post-tests, comparing each treatment concentration with the negative control group (each run on each day). The percentages of reduction (or increase) of viability, as described in the Results section, were taken from the relative motility index by using the formula % reduction = (1 − RMI) × 100 (for example, an RMI of 0.2 corresponds to 80% of viability reduction).

Microscopical observation of motility and morphological changes of the TTy were also employed in all assays as before [33,34,35,36]. The observation was performed with an inverted microscope (Primo Vert, Carl Zeiss, Oberkochen, Germany) and images were taken using a digital video camera (AxioCam ERc5c, Carl Zeiss, Oberkochen, Germany).

## 5. Conclusions

This study provides laboratory evidence of the in vitro effect of the STL estafietin, as well as its parent extract of *S. alpina* var. *alpina* on *M. vogae*, an experimental cestode model. The early and potent cestocidal effects observed suggest that *Stevia* extracts and compounds can be explored as new therapeutic alternatives to be applied alone or in combination with classical medicines such as albendazole to treat neglected tropical diseases caused by these types of parasites. Future research studies employing in vivo models of the zoonotic parasite *E. granulosus sensu lato* will further corroborate and expand our current findings on *M. vogae*.

## Data Availability

The original contributions presented in the study are included in the article, further inquiries can be directed to the corresponding authors.
